# Tuning of Schottky Barrier Height at NiSi/Si Contact by Combining Dual Implantation of Boron and Aluminum and Microwave Annealing

**DOI:** 10.3390/ma11040471

**Published:** 2018-03-22

**Authors:** Feng Sun, Chen Li, Chaochao Fu, Xiangbiao Zhou, Jun Luo, Wei Zou, Zhi-Jun Qiu, Dongping Wu

**Affiliations:** 1 State Key Laboratory of ASIC and System, Fudan University, Shanghai 200433, China; 15210720083@fudan.edu.cn (F.S.); 16110720066@fudan.edu.cn (C.L.); 12110720010@fudan.edu.cn (C.F.); 12212020051@fudan.edu.cn (X.Z.); 2 Key Laboratory of Microelectronic Devices and Integrated Technology, Institute of Microelectronics, Chinese Academy of Science, Beijing 100029, China; luojun@ime.ac.cn; 3 Process Application, Applied Materials, Inc., Gloucester, MA 01930, USA; Wei_Zou@amat.com

**Keywords:** Schottky barrier height, SB-MOSFET, dopant segregation, microwave annealing, dual implantation

## Abstract

Dopant-segregated source/drain contacts in a p-channel Schottky-barrier metal-oxide semiconductor field-effect transistor (SB-MOSFET) require further hole Schottky barrier height (SBH) regulation toward sub-0.1 eV levels to improve their competitiveness with conventional field-effect transistors. Because of the solubility limits of dopants in silicon, the requirements for effective hole SBH reduction with dopant segregation cannot be satisfied using mono-implantation. In this study, we demonstrate a potential solution for further SBH tuning by implementing the dual implantation of boron (B) and aluminum (Al) in combination with microwave annealing (MWA). By using such a method, not only has the lowest hole SBH ever with 0.07 eV in NiSi/n-Si contacts been realized, but also the annealing duration of MWA was sharply reduced to 60 s. Moreover, we investigated the SBH tuning mechanisms of the dual-implanted diodes with microwave annealing, including the dopant segregation, activation effect, and dual-barrier tuning effect of Al. With the selection of appropriate implantation conditions, the dual implantation of B and Al combined with the MWA technique shows promise for the fabrication of future p-channel SB-MOSFETs with a lower thermal budget.

## 1. Introduction

Schottky-barrier metal-oxide semiconductor field-effect transistors (SB-MOSFETs) [1], also known as metallic source/drain (S/D) MOSFETs (MSD-MOSFETs) [2], have emerged as potential candidates for replacing the conventional p–n junction in an S/D contact. SB-MOSFETs exhibit (1) lower parasitic resistance and capacitance; (2) a shallower junction depth; and (3) a shorter response time than conventional MOSFETs [1,3]. However, to further improve their performance, the effective Schottky barrier height (SBH) of SB-MOSFETs must be reduced to below 100 meV to overcome problems such as low drive currents and high contact resistance during scaling [4,5,6]. To meet this objective, various metal silicides have been introduced, including nickel silicide because of its low formation temperature and limited Si consumption [7]. Dopant segregation (DS) [8] schemes aiming at SBH interfacial property regulation have also been implemented, including the use of silicide as a diffusion source (SADS) [7,9,10], silicide-induced dopant segregation (SIDS) [7,11], and other modified schemes [12].

For n-channel SB-MOSFET S/D contacts, an electron SBH (φ_bn_) of 0.07 eV has been achieved in NiSi/p-Si contacts via arsenic (As) DS [7,11]. Other dopants, such as sulfur [13], selenium [14], and antimony [15], have also been employed for effective φ_bn_ tuning using various mechanisms. For p-channel SB-MOSFET S/D contacts, the extreme hole SBH (φ_bp_) achieved remains 0.11 eV in NiSi/n-Si contacts via boron (B) DS [7,12]. Because of the solubility limit of B in silicon, there is little room to further exploit the capacity for SBH regulation using mono-implantation of B [11]. It has been reported that aluminum (Al) DS can reduce the φ_bp_ value to 0.11 eV in NiSi/p-Si contacts [16,17,18] and that indium DS can reduce this value to 0.16 eV [19]. According to Shannon, a p-type dopant could also be used to tune φ_bp_ in an n-type substrate and vice versa [20]. Hence, Al is an ideal alternative for introduction into a p-channel metal S/D to achieve further φ_bp_ reduction.

The microwave annealing (MWA) process has gradually been accepted as an alternative annealing method because of its various beneficial properties, such as selective annealing [21], reduced diffusion [22], and higher activation of the dopant at a relatively lower temperature [23] compared with conventional thermal annealing methods, such as furnace annealing and rapid thermal annealing (RTA). The capacity toward effective SBH regulation in a DS NiSi/Si contact was improved when using 600-s MWA instead of 600-s RTA at a lower temperature [11]. However, to improve the competitiveness of MWA for SBH tuning, the duration of MWA should be reduced to achieve further reduction of the thermal budget. Previous works have shown that microwave absorption can be enhanced in silicon by using heavier ions (B/As) or Ge/Si pre-amorphization implantation [24], which would lead to further defect generation [23]. Therefore, a feasible strategy to enhance the absorption of microwaves is to increase the defect density by implanting an additional dopant, for which Al is an ideal candidate.

In this work, we propose a novel solution to achieve further effective SBH tuning and MWA absorption enhancement by implementing dual implantation of B and Al with varying implantation energies. The SIDS method was used for further thermal budget control [7,11], and the corresponding SBH regulation mechanisms were investigated.

## 2. Materials and Methods

Four-inch n-type silicon wafers (100) with resistivity of 1–10 Ω·cm were used for the device fabrication. Following the RCA clean to remove any contaminants, a 200-nm-thick SiO_2_ isolation layer was grown on each substrate using low-pressure chemical vapor deposition. The active regions were defined using 75-μm-radius circular patterns. B with an energy of 1 keV and Al with an energy ranging from 2 to 10 keV were sequentially implanted into the substrates at equal doses of 1 × 10^15^ cm^−2^ with a Varian High Current Implanter (Applied Materials, Inc., Gloucester, MA, USA). The detailed implantation conditions are listed in Table 1. Next, an HF dip was used to remove the native oxide layer, and a 40-nm-thick Ni layer was sputter-deposited onto the surface. The wafers were then sliced into square samples with dimensions of 22 mm × 22 mm and divided into four groups named N1, N2, N3, and N4 according to their implantation conditions. MWA was performed in an AXOM-200 (DSG Technologies, Inc., Santa Clara, CA, USA) octagonal MWA chamber at 5.8 GHz. Each sample was separately heated in an N_2_ atmosphere for 60 s at various MWA power conditions (1120, 1750, 2590, 2800, and 3500 W). The susceptor-assisted method [22,25,26] was employed to aid the heating of the substrate during the MWA process. After the annealing process, residual Ni was removed by immersing the samples in a 2:1 H_2_SO_4_:H_2_O_2_ solution at a constant temperature of 120 °C for 600 s.

## 3. Results and Discussion

**Figure 1:**
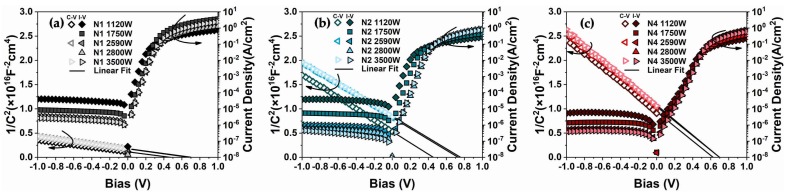
Current–voltage (I–V) and 1/C^2^−V characteristics of (**a**) N1, (**b**) N2, and (**c**) N4 samples.

**Figure 2:**
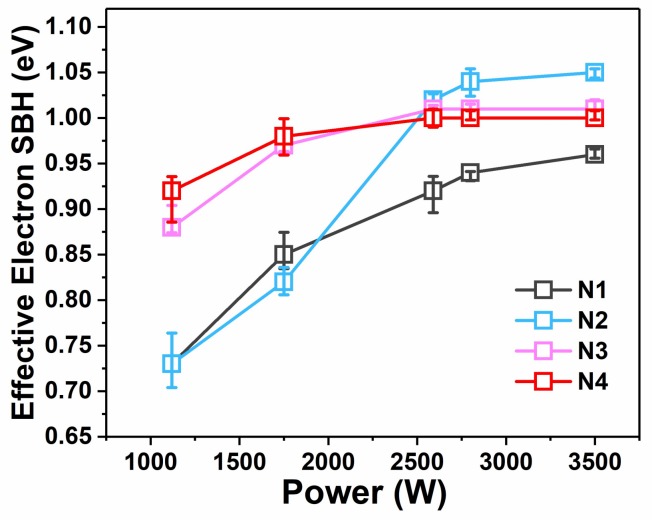
Effective φ_bn_ as a function of MWA power. SBH: Schottky barrier height.

To characterize the Schottky diodes, the current–voltage (I–V) and capacitance–voltage (C–V) methods were both implemented. Using standard C–V measurements for SBH extraction [9,11], the C–V curves were converted into 1/C^2^−V curves, where the intercept and slope of the linear fit were used to determine the SBH of the diodes [27,28]. In extracting the SBH for an n-type substrate, the SBH is denoted as φ_bn_. Because φ_bn_ + φ_bp_ = 1.12 eV, an increase in φ_bn_ means a decrease in φ_bp_ and vice versa. Figure 1 presents the I–V and 1/C^2^−V characteristics classified by implantation conditions. The extracted φ_bn_ values are listed in Table 1 and depicted in Figure 2. As shown in Figure 1a–c, a monolithic trend of decreasing reverse currents with increasing MWA power was observed, which is similar to the trend observed in our previous research [11]. This tendency indicates a positive correlation between the SBH and microwave power and that more thorough activation and segregation of dopants likely occurs under higher-power microwave radiation. Moreover, the apparent reduction of the reverse currents in the samples with Al implantation suggests that the dual-implanted diodes exhibited an improved ability toward φ_bn_ elevation compared with N1, which is in agreement with the SBH results presented in Figure 2. The high values of φ_bn_ beyond 1.02 eV obtained for the dual-implanted specimens surpass extreme values in the literature obtained using single B implantation [7,12]. In other words, we achieved sub-0.1 eV φ_bp_ values via dual implantation for a NiSi/n-Si contact, which is ideal for a p-channel SB-MOSFET. This phenomenon can be attributed to the segregation of Al at the silicide/substrate interface, which enables the saturation limits of SBH regulation by B implantation to be overcome, such that the limit of dipoles generation at the interface is confined by the solubility of B in silicon. Further details of this regulation scheme will be discussed in the following sections.

**Figure 3:**
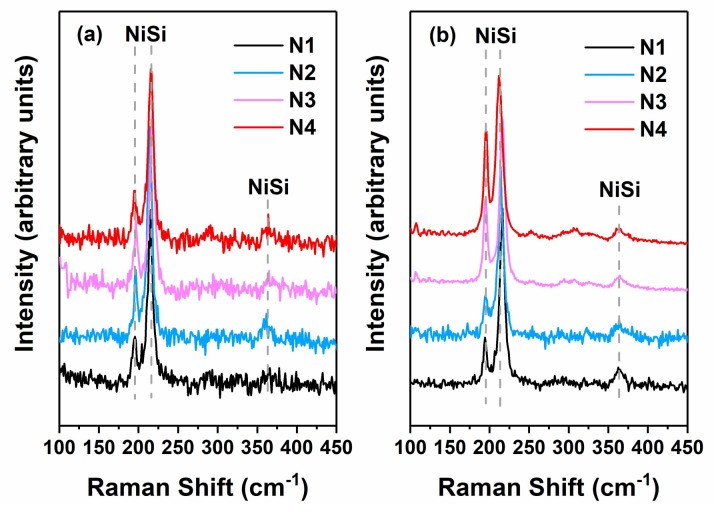
Raman spectra of samples prepared using MWA powers of (**a**) 1120 W and (**b**) 3500 W.

Additionally, an extended range of the rectification ratio [16] for the N2 samples was observed, as shown in Figure 1a, whereas the range of the N4 diodes was lower. This phenomenon is consistent with the 1/C^2^−V characteristics, indicating that φ_bn_ increased to various degrees upon increasing the MWA power (from 0.73 to 1.05 eV for N2 and from 0.93 to 1.00 eV for N4)**.** As shown in Figure 2, the effective SBH curves of the dual-implanted diodes converged below 2590 W and diverged again above this value. For lower microwave power (1120 W), the samples with higher Al implantation energy exhibited reduced φ_bn_ tuning ability than those with lower implantation energy. At higher MWA power (3500 W), the opposite trend was observed. These results imply an inverse relationship between the Al implantation energy and φ_bn_ tuning ability. To further investigate this phenomenon, Raman spectra of the samples prepared using MWA powers of 1120 and 3500 W were obtained. As shown in Figure 3a,b, obvious NiSi peaks [11,29] were detected in all the selected samples, indicating conversion of the nickel silicides into monosilicides. As the silicides at both ends of the MWA power range were verified to be NiSi, it is reasonable to conclude that all the silicides of the diodes in that range were monosilicides. Therefore, the SBH tuning scheme of dual-implanted diodes shows no relation with the nickel silicide phase, thereby necessitating further investigations. Moreover, the peak annealing temperature during the MWA process was determined to be below 344 °C, which is far below the lowest record of 400 °C using conventional thermal annealing methods [30], even when accounting for measurement errors produced by the infrared pyrometer [11]. Previous studies have demonstrated that the lower annealing temperature of the MWA process results from its unique non-thermal effect, which reduces the bonding activation energy during the recombination process [23,31]. Thus, a non-thermal effect could play a crucial role in reducing the temperature during nickel silicide formation.

**Figure 4:**
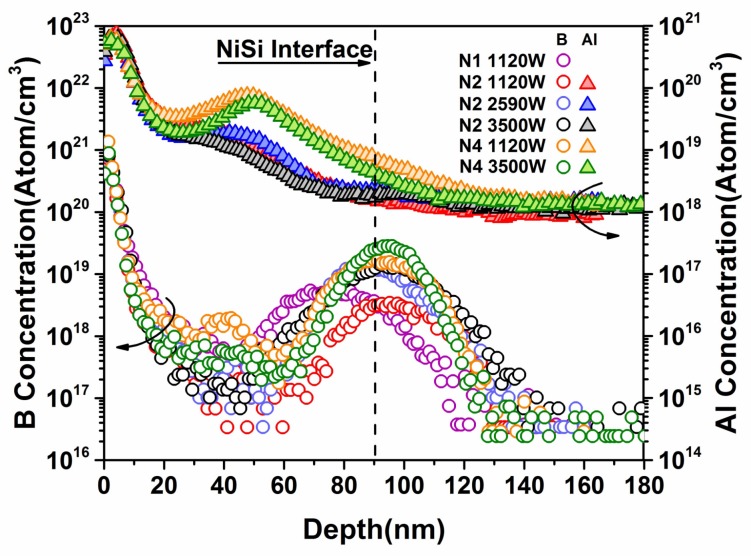
Separated SIMS (secondary-ion mass spectroscopy) depth profiles of Al and B in selected samples.

To further explore the SBH tuning mechanism of MWA dual implantation, secondary-ion mass spectroscopy (SIMS) was applied. Figure 4 presents the depth profiles of B and Al in the N1, N2, and N4 samples. The NiSi layer of the N1 samples (80 nm) was relatively thinner than that of the dual-implanted samples (90 nm). Based on the Raman results that the silicides have been confirmed as monosilicides, the thickness difference presumably arose from the occasional non-uniform deposition of Ni during the sputtering, which resulted in a rough interface after annealing. The distribution patterns of B and Al in Figure 4 are distinct, which can be attributed to the lower solubility of B in silicides [7], which resulted in a more protruding profile. In contrast, for Al, no obvious segregation peaks were detected at the NiSi/Si interface, which is similar to the observation by Sinha that the distribution curves tend to be more flattened with higher doses of Al, as the solubility of Al in NiSi (~6 × 10^20^ cm^−3^) is higher than that in Si (~2 × 10^19^ cm^−3^) [32]. As the dose of Al in our work (1 × 10^15^ cm^−2^) was even higher than that in Sinha’s work (2 × 10^14^ cm^−2^), Al presented a higher concentration at the interface, resulting in an even more covered segregation peak. The Al peak for the N4 samples at a depth of approximately 50 nm is assigned as the as-implanted Al peak in Si [33]. In addition, the long diffusion length in silicon is partly attributed to the diffusivity enhancement of Al by extrinsic B doping [34]. The disparity of the peak concentration at the interface between the dopants was credited to the solubility of B in Si exceeding that of Al [35].

Furthermore, when examining the samples prepared with an MWA power of 1120 W, a complex pattern of the concentrations of B and Al at the interface was observed. Compared with N1, N4 had a higher concentration of B at the interface, suggesting improvement of the segregation of B with the additional Al implantation. Comparison of N1 and N2 revealed that the accumulated concentration of both dopants at the interface was not sufficiently high to implement effective SBH tuning, with the φ_bn_ value remaining at 0.73 eV, which was same as the original SBH result of NiSi/Si contact [7]. Potential mechanisms driving this behavior need to be investigated. First-principles calculations indicate that dopants’ substitution of Si atoms approximately within the first Si monolayer from the interface of NiSi/Si could stimulate dipoles for band bending and SBH regulation [7,11,36]. Therefore, this variation may be related to the activation of dopants at the interface. Considering the implantation energy difference of Al, we believe various degrees of amorphization may lead to different levels of dopant activation in both mono- and dual-implanted diodes. Compared with the N2 samples with the implantation energy of 2 keV, the N4 samples were characterized by heavier and deeper amorphous regions because of the higher implantation energy of Al at 10 keV, which produced a projected range of approximately 20 nm [37]. The mean projected range can also be determined from the location of the first crest of Al in Figure 4, which appeared at the same position as the as-implanted Al peak. Given the deeper amorphous region produced by the higher implantation energy of Al, where strong rotation of dipoles occurs in response to the alternating electromagnetic field, the dielectric polarization loss effect was greatly induced [23], thereby enhancing absorption of microwaves during the MWA process. Combined with the intensified non-thermal effect reducing the dopant activation energy, the dopants were activated to a large extent. In contrast, for the N2 sample, the projected range of implanted Al with an implantation energy of 2 keV remained the same as that of implanted B with an implantation energy of 1 keV (6 nm) [37]. This shallower amorphous layer was rapidly consumed during the formation of nickel silicide. It has been reported that both the dielectric polarization loss effect and non-thermal effect vanish in silicon after the defects are fully repaired [23]. Therefore, sufficient microwave absorption could not be induced in N2 to enable the activation of the dual dopants, resulting in an insignificant non-thermal effect. Figure 4 also shows that the peak concentrations of B in both N1 and N2 were far below the saturation point.

A reversed correlation must be clarified when the MWA power reached 3500 W. As shown in Figure 4, higher concentrations of B and Al were segregated at the interface for N4 than for N2, even resulting in a narrower width of the segregated region of B. However, N2 showed better regulation toward φ_bn_ elevation, achieving a value of 1.05 eV, which is 50-meV higher than that obtained for N4. To obtain a better understanding of the aforementioned reversed correlation between φ_bn_ and the Al implantation energy, the N2 sample prepared using an MWA power of 2590 W was included for comparison. Except for being inadequately activated in the N2 samples prepared using MWA powers below 2590 W, no differences were observed in the distributions of B in the selected samples. However, an obvious discrepancy was observed between N2 and N4 as shown in Figure 4: a higher implantation energy of Al resulted in a higher Al concentration at the interface accompanied by a higher density of Al remaining in the NiSi film. Previous studies have indicated that Al has a dual-barrier tuning effect [18,38], including reducing φ_bn_ when incorporated with nickel silicide [39,40,41] and reducing φ_bp_ at NiSi/Si interfaces. It has been reported that the incorporation of Al in NiSi could reduce the metal work function of NiSi by up to 400 meV [39]. This finding was further confirmed by implanting carbon before Al combined with DS methods, which suppressed the diffusion of Al and sharpened its φ_bn_ reduction effect [38]. Thus, we attribute the aforementioned reversed tendency of SBH tuning with Al implantation energy to the different Al contents in NiSi, which inordinately affected the effective elevation of the SBH.

**Figure 5:**
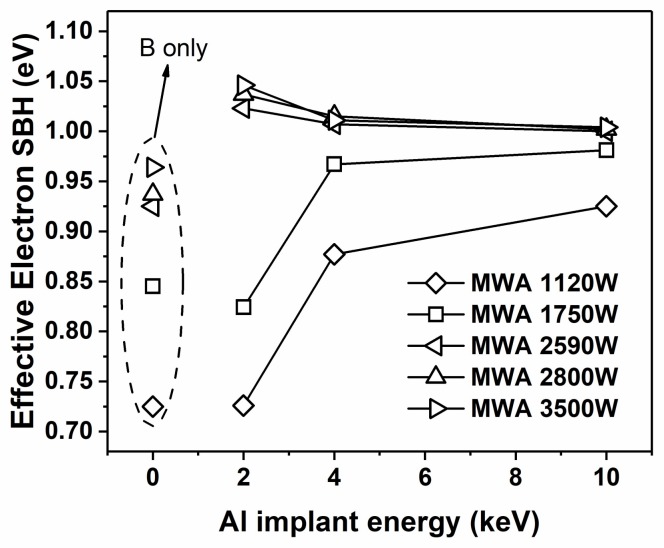
φ_bn_ tuning schemes of dual implantation associated with Al implantation energy. The points encircled by the dashed line represent samples implanted with B only. The squares represent the first stage, and the triangles represent the second stage.

Specific details about the SBH regulation schemes of additional implanted Al are presented in Figure 5. First, for MWA powers under 2950 W as shown in Figure 5, because Al was already saturated at the interface, no further concentration enhancement could be achieved by further increasing the power. Counterbalanced by the φ_bn_ reduction from Al in NiSi, Al showed weak control over φ_bn_ elevation. Effective SBH tuning is primarily affected by activated B at the interface. In addition, a higher implantation energy of Al also contributed to stronger activation and segregation of B for a deeper amorphous region. Thus, N4 showed better regulation toward effective φ_bn_ elevation than N2. Second, as the MWA power increased above 2950 W, as shown in Figure 5, B was adequately activated and segregated such that increasing the MWA power alone could not result in remarkable improvement. Combined with the highly segregated B, the φ_bn_ elevation capacity of Al started emerging. For the N2 sample, a φ_bn_ of 1.05 eV was achieved. However, although the tendency of draining Al in NiSi with increasing MWA power was observed in both N2 and N4, the effect of φ_bn_ elevation of Al remained insufficiently intense to overcome the φ_bn_ reduction effect in N4. As observed in Figure 4, compared with N2, substantial accumulation of Al still occurred above the interface in N4 after diffusion with promoted microwave radiation, which presumably affected SBH regulation [42]. In our case, with an MWA power of 3500 W, the calculated dose of Al from 50 nm above the interface and to that varied from 3.92 × 10^13^ cm^−2^ (N2) to 1.33 × 10^14^ cm^−2^ (N4) depending on the Al implantation energy of the device. This difference most likely resulted in an effective reduction of the work function of N4 for NiSi. Thus, an inconspicuous effect of φ_bn_ elevation was observed for N4 despite the higher concentration of Al at the interface.

Therefore, taking the two stages into consideration, the trade-off between the implantation energy and MWA power was attributed to the contradiction between the reduction and elevation of φ_bn_ of Al as well as that between the concentration of Al remaining in NiSi and that accumulated at the NiSi/Si interface. To overcome this contradiction and achieve higher φ_bn_ with lower MWA power, a thinner film of silicide [16] or a lower dose of Al [18] could be employed to sharpen the segregation peak at the NiSi/Si interface in future applications.

## 4. Conclusions

By implementing B and Al dual implantation, we demonstrate a potential solution for further effective φ_bp_ reduction to sub-0.1 eV levels and MWA absorption enhancement in NiSi/n-Si contacts. We report the lowest φ_bp_ achieved thus far of 0.07 eV at 500 °C (3500 W) in a NiSi/n-Si contact and a φ_bp_ of 0.2 eV at sub-350 °C (1120 W). Compared with 600-s MWA using mono-implantation, a compressed microwave annealing duration of 60 s was also achieved via the dual-implantation scheme. The SBH tuning schemes of the dual-implanted diodes were investigated, and higher energy of Al appears to induce microwave absorption, thereby resulting in high dopant activation and segregation. In addition, we explored the trade-off between the implantation energy of Al and MWA power in dual-implanted diodes. The contradiction of the reduction of the SBH of Al in NiSi and its elevation at NiSi/Si interfaces could potentially be solved by applying thinner silicides or a lower dose of Al. Benefiting from the unique properties arising from MWA, various combinations of dopants and triple-, quad-, or multi-implantation schemes accompanied by a lower thermal budget could be developed to achieve further SBH regulation.

## Figures and Tables

**Table 1:** Effect of implantation conditions and microwave annealing (MWA) power on effective φ_bn_.

Sample No.	Implant Species		MWA Power				
	B	Al	1120 W	1750 W	2590 W	2800 W	3500 W
	Implant Energy (keV)		φ_bn_ (eV)				
N1	1	-	0.73	0.85	0.92	0.94	0.96
N2	1	2	0.73	0.82	1.02	1.04	1.05
N3	1	4	0.88	0.97	1.01	1.01	1.01
N4	1	10	0.92	0.98	1.00	1.00	1.00
